# Reconstruction of 3D topographic landscape in soft X-ray fluorescence microscopy through an inverse X-ray-tracing approach based on multiple detectors

**DOI:** 10.1038/s41598-022-24059-y

**Published:** 2022-11-22

**Authors:** Matteo Ippoliti, Fulvio Billè, Andreas G. Karydas, Alessandra Gianoncelli, George Kourousias

**Affiliations:** 1 Elettra — Sincrotrone Trieste, S.S. 14 km 163,5 in Area Science Park, Basovizza, 34149 Trieste, Italy; 2 Institute of Nuclear and Particle Physics, National Center for Scientific Research “Demokritos”, 153 10 Agia Paraskevi, Athens, Greece

**Keywords:** Computational science, Particle physics, Imaging techniques, Microscopy

## Abstract

The study of X-ray fluorescence (XRF) emission spectra is a powerful technique used in applications that range from biology to cultural heritage. Key objectives of this technique include identification and quantification of elemental traces composing the analyzed sample. However, precise derivation of elemental concentration is often hampered by self-absorption of the XRF signal emitted by light constituents. This attenuation depends on the amount of sample present between the radiation source and detection system and allows for the exploitation of self-absorption in order to recover a sample topography. In this work, an X-ray-tracing application based on the use of multiple silicon drift detectors, is introduced to inversely reconstruct a 3D sample with correct topographical landscape, from 2D XRF count rates maps obtained from spectroscopy. The reconstruction was tested on the XRF maps of a simulated sample, which is composed of three cells with different size but similar composition. We propose to use the recovered 3D sample topography in order to numerically compute the self-absorption effects on the X-ray fluorescence radiation, thereby showing that a quantitative correction is possible. Lastly, we present a web application which implements the suggested methodology, in order to demonstrate its feasibility and applicability, available at: https://github.com/ElettraSciComp/xrfstir.

## Introduction

X-ray fluorescence (XRF) spectroscopy is a versatile and well-established investigation tool for qualitative detection and quantification of elemental distributions within different types of samples^1^. As such, this technique finds a wide range of applications, spanning from fields such as archeology^2^, cultural heritage^3,4^, geology^5^ and biology^6,7^. Systems that are based at synchrotrons often constitute the most advanced technological examples of these applications, having at their disposal a series of features, such as variable spot size below the micrometer and high particle flux^8,9^, that allow for finer analysis of complex samples.

The XRF radiation, which is generated within the constituent atoms of a sample by the photoelectric absorption of the incident beam, is emitted isotropically in every direction. Despite this characteristic behavior, the actual amount of XRF radiation emitted from a point source within the sample, may not be detected evenly among the available silicon drift detectors (SDD). This inconsistency in count rates can be attributed mainly to the self-absorption of the XRF radiation by the sample itself, which in turn depends on the difference in path length encountered by the XRF photons when traveling from the production point within the sample to different SDDs. In other words, the surface topography and the orientation of SDDs with respect to the sample surface and incident beam direction is responsible for modulating the XRF signal observed by the single detectors^10^. Depending on the severity in path length differences, the self-absorption effect could yield misleading results on the actual mass and concentration of the element being examined^11^. However, no straightforward solution has yet been found, due to the fact that there is no explicit way to decouple the absorption effects due to the sample topography and those due to its composition^12,13^.

Over the years, different attempts have been made to introduce data analysis techniques capable of modeling XRF behavior within the scanned samples, in order to also provide a basis for quantitative information retrieval and correct for the self-absorption effect. For example, Monte Carlo algorithms have been implemented in order to simulate the emission of XRF radiation and its detection^14–16^. Alternatively, data analysis methods have also been implemented to correct for the angular dependence directly on the two-dimensional (2D) XRF maps that are collected by the SDDs, through the use of the Fundamental Parameter (FP) Method^17–19^. In particular, Trojek 2011 successfully demonstrated the application of the FP Method to reconstruct the 2D surface relief map of a metallic object and reduce the effects of self-absorption on its XRF map^17^. This was achieved through the use of a single detector and knowledge of the angles between the source, the sample and the detector. However, this method does not provide a three-dimensional (3D) way to analyze the self-absorption effect at various depths, focusing primarily on the surface of the object and may not be appropriate for all scanned samples.

Steps towards a 3D characterization and correction of the problem, have been taken in more recent years^20,21^. In particular, in a work by Malucelli et al.^19^ the authors implemented a multimodal approach employing XRF Microscopy, Scanning Transmission X-ray microscopy (STXM) and Atomic Force Microscopy (AFM) on top of the FP method. Here, for each pixel in the 2D XRF maps, the absorption of both the incident beam and XRF radiation, is calculated by integrating the absorption term formulated in the FP method over the sample’s thickness derived from the AFM^22^. The density map, also required in this process, is then derived from the STXM data. By adopting this approach, the authors were successful in retrieving quantitative maps of molar concentration of different elements contained within two cell types from information coming from 8 detectors pertaining to the Low Energy XRF (LEXRF) system present at the TwinMic beamline Sincrotrone Trieste (Trieste, Italy)^23^. However, the implementation of such multi-modal approaches may be impractical to adopt, as it requires different types of image data acquisitions with different positioning and resolutions to be carefully registered together. Furthermore, the self-absorption term pertaining to the XRF radiation, is calculated for each pixel by summing together the emission within the corresponding sample volume and considering only a single possible path to be undertaken by the emitted radiation to reach its respective detector. In this manner, it is not possible to fully exploit the 3D topographic information of the sample and calculate for each 3D emission voxel, all the possible paths leading the radiation to the detectors. As a consequence, the effective length of the paths traveled may be underestimated by not considering gaps produced by valleys and hills in the topography, resulting in an inaccurate quantification of the attenuation.

The presented study builds on the findings of a previous work by Billè et al.^11^, in which an XRF simulation framework was presented, to verify the effects of the self-absorption artifact on the elemental distribution of XRF imaging of cells. It was shown how strongly the topographical effects can dampen the count rates of fluorescence emission lines pertaining to light elements such as Carbon (C), Nitrogen (N) and Oxygen (O). In the current work, we present a ray-tracing based method to inversely reconstruct a 3D sample with its topographical landscape, simply from 2D XRF maps acquired on multi-detector systems, together with an STXM map, an average density value and a maximum thickness value for the sample. Once the 3D sample structure has been reconstructed and its similarity with the actual simulated structure has been established, we propose to use it in order to numerically calculate the self-absorption effects exerted by the sample on the XRF radiation at a 3D level and show that a quantitative correction is possible.

## Methods

We introduce a novel 3D Inversion Reconstruction (IR) algorithm, which derives a 3D sample topography starting from 2D XRF images. The IR can be summarized into 3 main steps. First, we define a numerical simulation approach that from a 3D sample of known shape and composition, derives the corresponding 2D XRF maps that would be collected during a XRF experiment. Secondly, through the FP Method^24^, we obtain the sample’s elemental composition information necessary to run the simulation directly from the 2D XRF maps. Lastly, an in-house developed optimization approach based on ray-tracing, is employed to probe an ensemble of possible 3D sample topographies, and isolate the one minimizing the L1 norm between the actual experimental XRF maps and the simulated ones.

### XRF simulation

To numerically resolve the absorption effects of the sample, we first characterize the data acquisition process for the SDD. The following considerations are based on the LEXRF detection system of TwinMic Beamline in Elettra Synchrotron^23^, comprising a set of 8 SDDs with an incident energy ${E}_{0}$ between 400 and 2200 eV. Analogous considerations can be made for similar systems carrying out low-energy XRF experiments.

**Figure 1:**
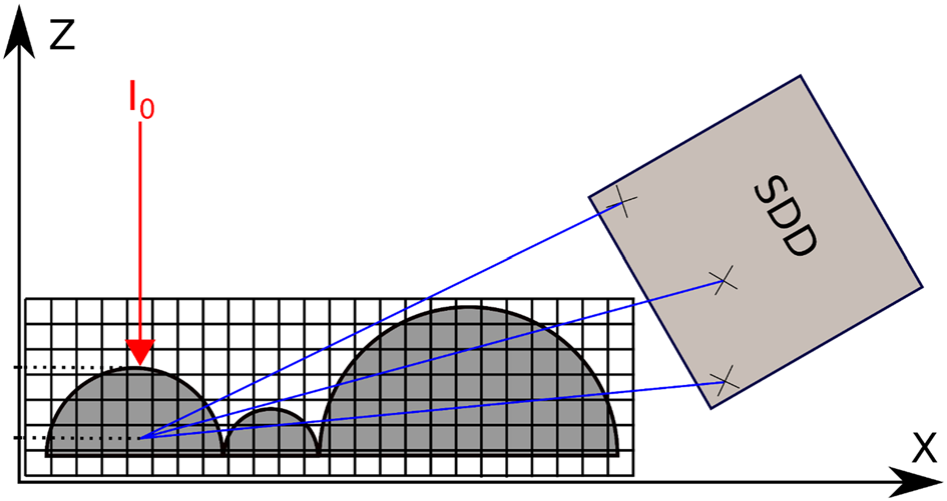
XRF microscopy acquisition setup for a single SDD. The incident beam ${I}_{0}$(red arrow) invests the sample parallel to the z-axis and travels a distance z before being absorbed, thereby isotropically producing XRF radiation, part of which is directed towards the SDD (blue arrows).

Figure 1 shows a typical XRF setup for one SDD. The incident radiation is aligned parallel to the z-axis of the sample’s frame of reference, investing the sample for a certain exposure time and with a specific spot, whose size defines the final image resolution of the XRF images. In this work the spot size was 1 μm^2^ isotropic and the incident intensity (${I}_{0}$) was $1{0}^{8}$ photons/μm^2^ integrated over the entire time of exposure, mimicking the conditions of a real experiment.

As radiation traverses the sample from its surface to the point of XRF radiation production, it is attenuated following the Beer–Lambert’s Law of Absorption ${I={I}_{0}e}^{-{\mu }_{s}({E}_{0})\rho z}$, where ${I}_{0}$ is the incident beam’s intensity, ${\mu }_{s}({E}_{0})$ represents the total mass attenuation coefficient of the sample at the beam energy ${E}_{0}$, $\rho$ represents the sample density and z is the traveled distance within the sample. As the incident radiation invests the sample, an absorption profile $AB{S}_{s}={\mu }_{s}({E}_{0}) \rho dz$ is generated along the z−axis. Here, we propose to derive the sample thickness map along the incident beam direction (z-map) in an iterative fashion, starting from a Scanning Transmission X-ray Microscopy (STXM) map.

Not all elements absorb the incoming radiation equally. To retrieve the fraction of the radiation which is absorbed via photoionization by every j-th element $AB{S}_{j}$, we need to multiply $AB{S}_{s}$ by the mass fraction ${w}_{j}$ and by the ratio of the photoionization cross-section of the j-th element (${\tau }_{j}({E}_{0})$) to ${\mu }_{s}$ at ${E}_{0}$^25^:

1 $${ABS}_{j}={ABS}_{s}\,\, {w}_{j} \frac{{\tau }_{j}\left({E}_{0}\right)}{{\mu }_{s}\left({E}_{0}\right)}={{w}_{j} \,\,\tau }_{j}\left({E}_{0}\right) \rho\,\, dz.$$

We then take into account the solid angle fraction covered by each detector $(\Omega /4\pi )$ and physical information related to the emission process for XRF line *i*, such as the fluorescence yield ${\omega }_{j}$, the transition probability ${p}_{ij}$ and the absorption jump ratio ${J}_{j}$, through the factor $Y_{ij}({E}_{0},{E}_{i})=(\Omega /4\pi ){\omega }_{j}\,{p}_{ij}\,{ J}_{j}$. We then compute the 3D fluorescence emission matrix $E{M}_{ij}$ of any element *j* in the sample^25^:

2 $${EM}_{ij}\left(z,{E}_{i}\right)={ABS}_{j} Y_{ij}\left({E}_{0},{E}_{i}\right){I}_{0}{e}^{-{\mu }_{s}\rho z}={{w}_{j}\,\, \rho\,\, \tau }_{j}\left({E}_{0}\right)\left(\frac{\Omega}{4\pi}\right){\omega }_{j} \,\,{p}_{ij} \,\,{J}_{j} \,\,{I}_{0} {e}^{-{\mu }_{s}\rho z} dz.$$

During the acquisition, photons emitted from the same voxel can take $\alpha$ different linear paths of length ${\delta }_{\alpha }$ towards the SDD. As it can be seen in Fig. 1, the differences in ${\delta }_{\alpha }$ depend on the portion of the sample traveled by the photon and in turn by the sample’s topography. The probability ${{P}^{\alpha }}_{T}$ that an XRF photon of energy ${E}_{i}$, directed along a single path α, will be able to arrive unabsorbed at the SDD’s interface, is as follows:

3 $${P}_{T}^{\alpha }={e}^{-{\mu }_{s}({E}_{i})\rho {\delta }_{\alpha }}.$$

We construct the 3D response matrix of any SDD (${XRF}_{3D}$) by multiplying Eq. (2) by Eq. (3) for every voxel and summing over all the possible $\alpha$ directions:

$${XRF}_{3D}=\sum_{\alpha =1}^{{N}_{\alpha }}\frac{{EM}_{ij}}{{N}_{\alpha }}{P}_{T}^{\alpha },$$

Here $N_{\alpha }$ represents the total number of α paths available to reach a given SDD. We divide $E{M}_{ij}$ by $N_{\alpha }$ to redistribute photons among all available paths. As fluorescence emission is an isotropic phenomenon, we expect the same number of photons to be emitted in all directions. $E{M}_{ij}$ is thus independent of the undertaken path and can be moved outside of the summation:

4 $${XRF}_{3D}={EM}_{ij}\sum_{\alpha =1}^{{N}_{\alpha }}\frac{{P}_{T}^{\alpha }}{{N}_{\alpha }}={EM}_{ij}\,\, K,$$

where *K* is a 3D matrix representing the self-absorption effects exerted by the sample. *K* represents an average of all ${P}_{T}^{\alpha }$ across all possible paths. Equation (4) is a numerical construction, built from the FP method, that can be employed to simulate the 3D experimental response of any SDD, having provided the necessary information on sample geometry and composition. Furthermore, one can move from the 3D response matrix for an SDD, to its 2D analogous ${XRF}_{2D}$, by summing Eq. (4) over the axis parallel to the incident beam:

$${XRF}_{2D}={\sum }_{z}{EM}_{ij} \,\,K.$$

Following, it will be shown how to obtain the sample’s compositional information from 2D experimental XRF maps. These will be then used to generate an ensemble of possible 3D sample topographies to be evaluated through an iterative optimization procedure based on ray-tracing and Eqs. (2) and (4).

### Deriving sample thickness map

We retrieve thickness information from the STXM data concurrently acquired with XRF data. Through approximate knowledge of the average sample density, maximum sample thickness and of the ranges of existence of both these quantities, it is possible to create an iterative procedure which at first generates a z-map according to the Law of absorption^19^, by considering the product of ${\mu }_{s}({E}_{0})\rho$ initially constant. Successively, the algorithm identifies pixels where the derived thickness changes abruptly from its nearest neighbors, by choosing a threshold according to the average rate of change observed throughout the whole sample. These pixels are then adjusted iteratively in terms of both thickness and density.

### Deriving compositional information for XRF simulation

The FP Method^19,25^ can be employed at a pixel level to retrieve the necessary compositional information, namely: ${w}_{j}$ and ${\mu }_{S}$ at the different ${E}_{i}$ considered. ${w}_{j}$ is calculated by first finding the mass of each element ${m}_{j}$:

5 $${m}_{j}=\frac{{C}_{i} S}{{I}_{0} Y_{ij}\left({E}_{0},{E}_{i}\right) {\tau }_{j}({E}_{0}) {K}_{FP}},$$

where S is the surface area of the pixel, ${C}_{i}$ represents the cumulative counts of the emission line *i* recorded in the XRF spectra, and ${K}_{FP}$ is an absorption correction factor related to both the incident and XRF radiation, as derived from the FP method:

$${K}_{FP}=\frac{1-{e}^{-\rho h\left({\mu }_{s}\left({E}_{0}\right)\mathrm{cosec}\left(\theta \right)+{\mu }_{s}\left({E}_{i}\right){\sum }_{\alpha =1}^{{N}_{\alpha }}\frac{\mathrm{cosec}({\varphi }_{\alpha })}{{N}_{\alpha }}\right)}}{\rho h\left({\mu }_{s}\left({E}_{0}\right)\mathrm{cosec}\left(\theta \right)+{\mu }_{s}\left({E}_{i}\right){\sum }_{\alpha =1}^{{N}_{\alpha }}\frac{\mathrm{cosec}({\varphi }_{\alpha })}{{N}_{\alpha }}\right)}=\frac{1-{e}^{-\rho h\left({\mu }_{s}\left({E}_{0}\right)\mathrm{cosec}\left(\theta \right)+{\mu }_{s}\left({E}_{i}\right){\langle \mathrm{cosec}(\varphi )\rangle }_{\alpha }\right)}}{\rho h\left({\mu }_{s}\left({E}_{0}\right)\mathrm{cosec}\left(\theta \right)+{\mu }_{s}\left({E}_{i}\right){\langle \mathrm{cosec}(\varphi )\rangle }_{\alpha }\right)},$$

where h is the total sample thickness along the z-axis for the pixel under consideration in the 2D XRF image*,* $\theta$ is the angle at which the incident beam invests the sample and $\varphi$ represent all the possible different α paths available to reach the SDD. In the last passage of the equation, the term ${\sum }_{\alpha =1}^{{N}_{\alpha }}\frac{\mathrm{cosec}({\varphi }_{\alpha })}{{N}_{\alpha }}$ has been substituted with ${\langle \mathrm{cosec}(\varphi )\rangle }_{\alpha }$. For most setups, like the one treated in this work, the incident beam is perpendicular to the sample and therefore the term $\mathrm{cosec}(\theta )=1$ and can be omitted.

Once all masses in a pixel are known, a single ${w}_{j}$ is calculated by dividing the mass of said element ${m}_{j}$ by the total mass ${m}_{s}$ as follows, ${w}_{j}={m}_{j}/{m}_{s}$. The maps for ${\mu }_{s}({E}_{0})$ and for ${\mu }_{s}({E}_{i})$, can then be calculated pixelwise, using the elemental mixture rule^26–29^:

6 $${\mu }_{s}\left({E}_{i}\right)={\sum }_{j}{w}_{j}{\mu }_{j}({E}_{i}),$$

which takes into account the mass absorption coefficients of the single elements ${\mu }_{j}$, known from universal tables, and ${w}_{j}$.

We propose to firstly retrieve the mass fractions ${w}_{j}$ by discarding ${K}_{FP}$ in Eq. (5). Successively, maps of ${\mu }_{s}({E}_{0})$ and ${\mu }_{s}({E}_{i})$ are calculated from Eq. (6) and employed by the ray-tracing IR algorithm to find a 3D topography. This topography attempts to minimize the L1 norm between the experimental XRF maps and the ${XRF}_{2D}$ maps obtained through simulation using the newly reconstructed sample and Eq. (4). At a second stage, we employ the reconstructed sample topography to calculate the emission matrix $E{M}_{ij}$, thereby attempting to retrieve the correct cumulative counts for each emission line *i*, before self-absorption takes place.

### Ray-tracing based inverse reconstruction

The presented IR algorithm exploits both complementary and redundant information from the multiple detectors available to infer a sample topography. The general idea is to:

1. iteratively derive a z-map from a STXM scan with the use of Beer–Lambert’s Law, an average density value and a maximum thickness value for the sample;
2. use the z-map to generate an ensemble of possible topographies. This is limited by a geometric constraint imposing that along the beam’s axis the sample is continuous and free from gaps;
3. verify the goodness of each sample topography by calculating Eq. (4) and confronting the result of each simulated ${XRF}_{2D}$ map with the actual data, by means of the L1-norm.

The z-map derivation is an iterative procedure that requires estimates of the average sample density and the maximum sample thickness we can expect. In the first iteration we associate the most absorbing pixels in the STXM with the maximum expected thickness. We then consider temporarily, the product of density and mass attenuation coefficient as constant for every pixel. In this way we can calculate a thickness map for all the remaining pixels. We then calculate over this thickness map the average slope present between each pixel and its nearest neighbors. This value is used as a threshold to select those pixels which seem to change thickness too abruptly with respect to the average change observed in the sample. All the identified pixels are then selected and their thickness value is replaced with the average thickness value of its nearest neighbors. At the same time the linear attenuation coefficient is re-calculated for these pixels from the STXM using the newly found thickness. A new iteration can now begin and repeat the whole process until completion.

**Figure 2:**
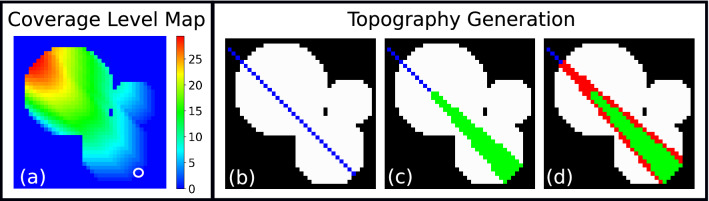
Coverage level map for the South-East SDD (**a**), showing the amount of sample in µm present between each pixel and the detector’s center. A first seed pixel, with minimal sample coverage (white circle) is picked. Other seed pixels are identified along the line (blue) connecting the SDD’s center with this first seed (**b**). The reconstruction process initializes through a first conical region of interest (**c**), which is resolved and fixed before moving onto the next region (**d**).

During the IR, each SDD reconstructs a topography independently of the other detectors. Subsequently, the information from the different detectors is merged together to yield a single 3D representation of the sample. Let us therefore consider a single SDD. Through the use of ray-tracing, each pixel in the XRF_2D_ maps is assigned a value that quantifies fraction present between it and the center of the detector’s face, as shown in Fig. 2a. We then select a seed pixel among the ones with the lowest amount of sample shielding from the detector (white circle) and we identify all the pixels lying on the line going through this seed and the detector’s center, as shown in Fig. 2b. We then pick a number of equally spaced pixels from this line (blue), in the direction moving away from the SDD which is placed in the South-East corner of the image. For each of these new pixels a conical region of interest (green) is identified, which encloses all the possible directions that an XRF photon can take to hit the SDD’s active surface as shown in Fig. 2c. Within this green region, we then need to identify all the possible geometrical permutations there can be in placing the sample. This is accomplished by firstly recovering the sample thickness map along z and expanding the sample in 3D, starting from a flat support in the xy plane and then growing each pixel along z. Secondly, all viable permutations are generated by rigidly translating each pixel in the green region along the z-axis in different combinations. The rationale behind this point is that among the generated topography permutations there is also the correct 3D topography, or at least a close representation. The only assumption made here to make a topography viable, is to have sample continuity along z. In other words, the rigid translations that bring about gaps along z between adjacent pixels in the 2D conical region of interest are not allowed. This is done in order to reduce the ensemble’s size and therefore ease the computational load.

Each 3D topography is then tested, by generating the elemental XRF_2D_ maps through Eq. (4) and computing the L1-norm map with respect to the corresponding XRF_2D_ maps from the experiment. A score is assigned to each candidate, which is equal to the pixel-wise sum of the L1-norm map. After all candidates have been validated, the lowest scoring topography is fixed in 3D for the green region and the whole process is repeated in the next conical region of interest in Fig. 2d (red region). This new conical ROI, will therefore partly contain an already resolved portion of sample (green region), rendering the complete reconstruction process along the blue line affordable in terms of computation. Once the reconstruction of a full blue line is finished, a new line is identified which covers different portions of the sample and the whole process is repeated until completion.

In order to speed up the reconstruction, two different parameters were introduced in the algorithm: the permutations threshold (δ) and the Inverse Reconstruction oversampling parameter ($I{R}_{os}$). Regarding the former, it determines the maximum number of candidate topographies that will be generated and tested inside the conical regions of interest shown in Fig. 2c,d. If for example, one such region has 10^4^ total permutations available, by setting δ equal to 10^3^, the system will take a permutation in every 10 for testing. In this process the candidate topographies are always sampled equidistantly from each other, in order to avoid selection biases. As for the latter parameter, thanks to coverage level maps such as the one in Fig. 2a, it is possible to establish for each pixel what is the most attenuated detector and normalize all coverage levels from the remaining detectors with this value. If the normalized coverage level value of a detector for a specific pixel is smaller or equal to the selected $I{R}_{os}$, then the pixel will be reconstructed for that detector, otherwise it will be excluded from the process ($I{R}_{os}=0$ closest detector only, $I{R}_{os}=1$ all detectors). The last step to be considered before the final single 3D sample can be derived, involves the merging of the different topographies computed independently from each detector. For each pixel, we set the normalized coverage level values associated to each SDD, as the weights of a weighted average. In this manner, the final 3D sample configuration is constructed in each pixel, by giving more weight to the detectors which are least attenuated by the sample. The presented algorithm was developed in-house with Python (Python Software Foundation) and the just-in-time compiler Numba^30^.

### Simulated sample

The sample simulated in this study consists of three hemispheres of radii 6, 9 and 12 $\mu m$ respectively to simulate cells of different sizes. The ${w}_{j}$ composition was based on the characteristic one of human colon carcinoma cells LoVo^19^, which consists of 61% C, 17% N and 16% O and average density value of 1.25 g/cm^3^. Three small regions of interest were chosen, in order to introduce an additional element, Magnesium (Mg), with a ${w}_{j}$ of 4%. As required assumptions, the sample’s elemental composition is considered homogeneous along the z-axis and also the sample’s thickness along the same axis is considered to be continuous with no interleaving gaps.

### Quantitative evaluations

$E{M}_{ij}$ In Eq. (2) describes the XRF photons in every voxel source, prior to self-absorption, allowing us to retrieve the mass of each element composing the sample. Calculating $E{M}_{ij}$ precisely involves knowledge of both the sample’s 3D structure and the ${w}_{j}$ of all the elements therein. The latter could be found by using the sample structure retrieved through the IR to compute Eq. (4) for different sets of mass fractions. The optimal set could then be chosen as the one minimizing the L1 norm between the ${XRF}_{2D}$ images produced by varying the ${w}_{j}$ sets, and the experimental XRF maps. However, the generation of such a system to fit ${w}_{j}$ goes beyond the scope of this work. Here we simply show that knowledge of a 3D structure, as reconstructed with the proposed algorithm, allows us to perform a quantitative correction of the elemental presence, provided the correct ${w}_{j}$ are known.

Two $E{M}_{ij}$ matrices were calculated using the same 3D sample structure recovered through the IR: one with the correct ${w}_{j}$ for all the elements ($E{M}^{c}$) and one with the incorrect ${w}_{j}$ using Eq. (5) by omitting ${K}_{FP}$ ($E{M}^{u}$). $E{M}^{c}$ and $E{M}^{u}$ are compared against the true emission matrix simulated ($E{M}^{s}$) via Eq. (2), obtained using the correct ${w}_{j}$ and the true 3D sample. The same comparison is carried out, on the ${XRF}_{2D}$ maps summed across all detectors, having defined the image generated with: the correct mass fractions and IR sample structure ($\Sigma XR{F}^{c}$); the incorrect mass fractions and the IR sample structure ($\Sigma XR{F}^{u}$); the correct mass fractions and the true 3D sample matrix ($\Sigma XR{F}^{s}$).

The statistical analysis consisted in determining the least square differences from the true images *(*$\overline{\Delta {EM}_{u}}$*, *$\overline{\Delta {EM}_{c}}$*, *$\overline{\Delta {XRF}_{u}}$*,* $\overline{\Delta {XRF}_{c}}$). All images were summed along the z-axis before the analysis, which was then carried out over 778 pixels constituting the entire 2D sample. Statistical significance in the difference of the mean values was assessed through a paired t-test, after verifying the normality assumption, through the 1-sample Kolmogorov–Smirnov test. Differences were considered to be significant for *p* < 0.001.

## Results

### 3D sample reconstruction

**Figure 3:**
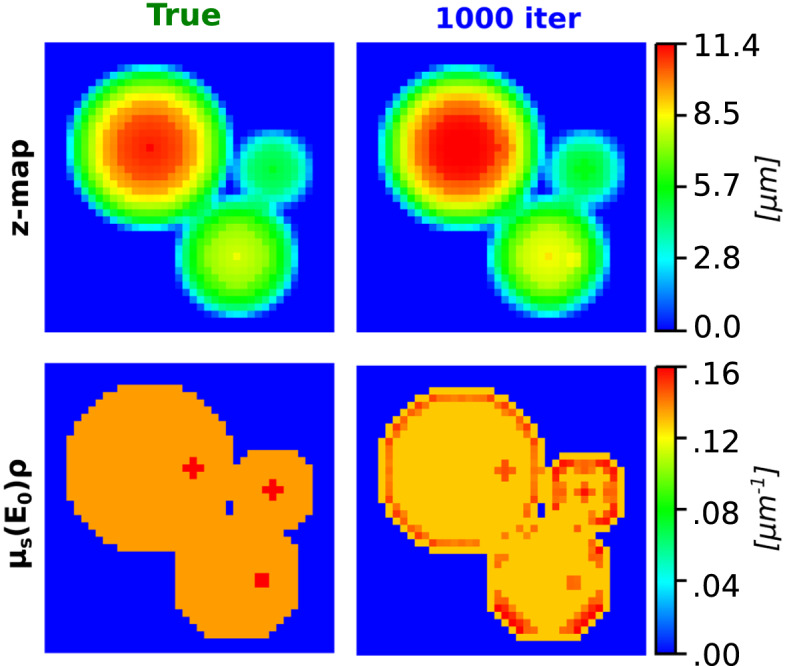
Comparison of z-map (top row) and ${\mu }_{s}({E}_{0})\rho$ (bottom row), between the ground truth (left column) and data recovered from STXM after 1000 iterations (right column).

All necessary compositional information, such as the mass fractions of the different elements ${w}_{j}$ present, the mass absorption coefficients of the elemental mixture making up the sample at the different line energies ${\mu }_{s}({E}_{i})$ and beam energy ${\mu }_{s}({E}_{0})$, were derived through Eqs. (5) and (6). The count rate maps $XR{F}^{s}$ pertaining to the ${K}_{\alpha }$ emission line of Carbon, were chosen as the target images for the optimizer of the ray-tracing IR algorithm. Carbon emission lines were chosen as an example since due to their low energy, they are most subject to self-absorption. Thickness information was successfully extracted from STXM data through the proposed iterative procedure, as can be observed in Fig. 3. After 1000 iterations the observed average percentage difference between the true z-map and the recovered one was − 3.4 ± 5.3%, while for ${\mu }_{s}({E}_{0})\rho$ the difference was 2.9 ± 5.5%.

**Figure 4:**
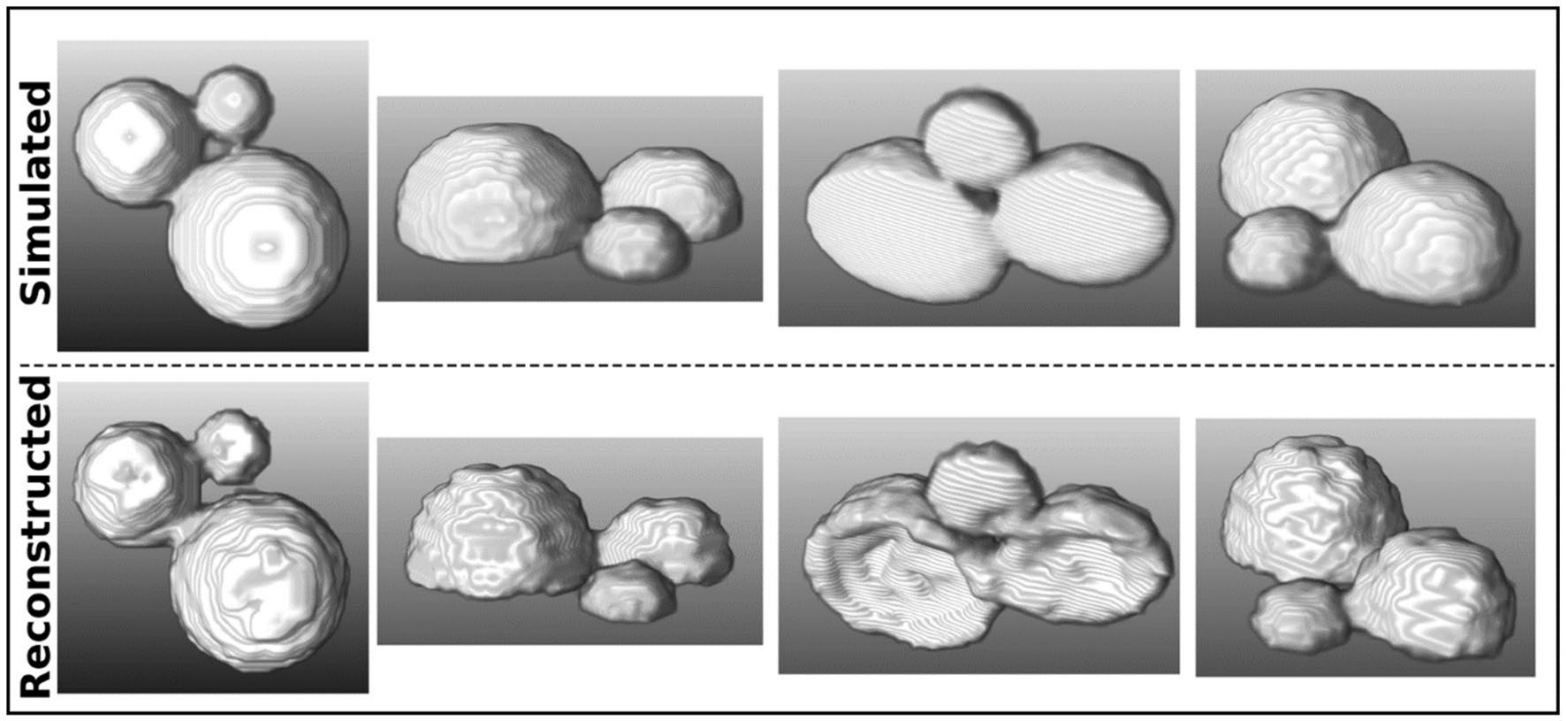
Comparison of simulated sample (top row) versus the reconstructed sample (bottom row).

Figure 4 shows the comparison between various profiles of the actual artificial sample (top row) and the reconstructed sample (bottom row). As it can be seen, most of the reconstructed sample already qualitatively shows a good resemblance with the actual sample in terms of topographic features such as the slopes and valleys present when moving from one hemisphere to another. The overall similarity of the two structures is also highlighted by the more quantitative evaluation of the volume percentage overlap which was found to be at 82% with $I{R}_{os}=0.2$ and 86% with $I{R}_{os}=1$.

### Cumulative counts correction

**Figure 5:**
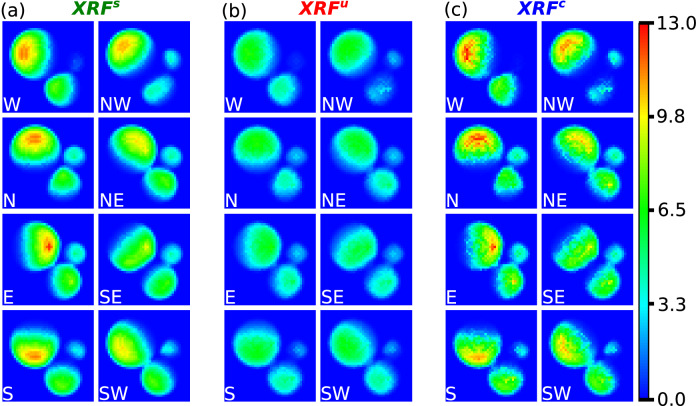
${XRF}_{2D}$ maps for the ${K}_{\alpha }$ line of C of the ${XRF}^{s}$ data (**a**), ${XRF}^{u}$ data (**b**) and ${XRF}^{c}$ data (**c**) respectively, displayed with the same logarithmic range. Each column displays images for all 8 SDDs comprising the LEXRF system, namely the West (W), North-West (NW), North (N), North-East (NE), East (E), South-East (SE), South (S) and South-West (SW) detectors.

After the retrieval of a 3D sample structure through the IR, the exact mass fractions of the elements present were employed in order to calculate ${XRF}_{3D}$ and ${XRF}_{2D}$ through Eq. (4) for all SDDs. This was done in order to quantitatively evaluate the effects of the absorption correction based on the newly acquired 3D sample structure, which is derived from inexact mass fractions directly from the XRF data. Figure 5a–c shows ${XRF}_{2D}$ maps of the cumulative counts for the ${K}_{\alpha }$ line of C for the $XR{F}^{s}$, $XR{F}^{u}$ and corrected data $XR{F}^{c}$ respectively, displayed with the same range. The $XR{F}^{u}$ and $XR{F}^{c}$ maps are both generated using the same structure from the IR. While the former are obtained via the inexact mass fractions through Eq. (5) by omitting the ${K}_{FP}$ term, the latter are obtained using the exact mass fractions. The simulated data on the other hand, is obtained by using the actual structure and mass fractions and is therefore used as the target to be achieved by the correction. As it can be seen, the maps from the corrected structure qualitatively show a good level similarity with the simulated data in terms of count rate, intensity and absorption features. The same does not hold true for the uncorrected data which deviates more strongly from the simulated images, especially in terms of count rates.

**Figure 6:**
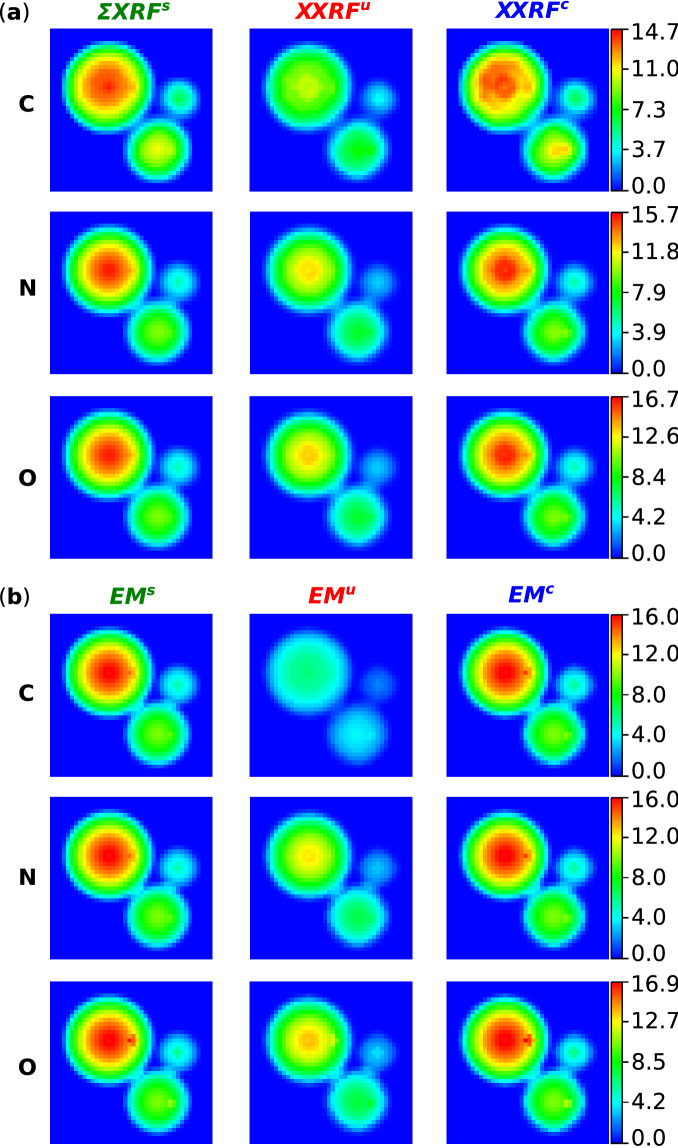
Sum of the count rates in logarithmic scale, across all the SDDs of the $\Sigma XRF$ maps (**a**) and of the ${EM}_{ij}$ matrices (**b**), for elements C, N and O included in the simulation, specifically for the line ${K}_{\alpha }$ and for the simulated, absorption uncorrected and absorption corrected data respectively.

Figure 6a shows the sum of the count rates across all the SDDs for C, N and O, specifically for the line ${K}_{\alpha }$ and for the $\Sigma XR{F}^{s}$, $\Sigma XR{F}^{u}$ and $\Sigma XR{F}^{c}$ data respectively. As it can be observed in the uncorrected maps, there is a global and substantial drop in the count rates associated with all the elements. By directly confronting the uncorrected images with the simulated ones it is evident that most of the count rates lost in the uncorrected data, predominantly come from the central part of the cells. On the other hand the corrected maps overall show count rates much closer to the simulated data. However, stronger deviation from the simulated intensities can also be observed in regions of the corrected maps that correspond to a stronger deviation of the reconstructed sample from the actual sample. Figure 6b shows the fluorescence emission matrices EM for line ${K}_{\alpha }$, and for the $E{M}^{u}$, $E{M}^{s}$ and $E{M}^{c}$ images of C, N and O, respectively. Similarly to Fig. 6a, we can appreciate the stronger resemblance between the corrected and simulated data over the uncorrected and simulated data. Once again, the uncorrected images systematically show lower count rates, especially as we move from the borders to the inner parts of the cells. It is also interesting to notice that in Fig. 6a, the ${\Sigma XRF}^{s}$ map of C shows a drop in signal within the central part of the largest cell facing the other two cells, which is not observed in the homologous $E{M}^{s}$ map.

The quantitative evaluation regarding the average least squares difference of the uncorrected and corrected maps from the simulated ones, was carried out on the $\Sigma XRF$ maps and $E{M}_{ij}$ matrices for line ${K}_{\alpha }$ of C, N and O and is reported in Table 1. All t-tests resulted to be statistically significant to the 1-sample Kolmogorov–Smirnov test and the paired sample t-test (p < 0.001). Confirming the visual inspection of Figs. 5 and 6a,b, the corrected images systematically demonstrated values close to the ground truth, both in the case of $\Sigma XRF$ (1.36 × 10^5^ ± 1.33 × 10^5^ for C, 1.52 × 10^5^ ± 1.35 × 10^5^ for N and 3.99 × 10^5^ ± 3.24 × 10^5^ for O) and in case of $E{M}_{ij}$ (2.27 × 10^4^ ± 1.45 × 10^4^ for C, 2.29 × 10^4^ ± 1.46 × 10^4^ for N and 5.73 × 10^4^ ± 3.65 × 10^4^ for O) images.

**Table 1:** Quantitative evaluation results.

	$\overline{\Delta \Sigma {XRF}_{u}}$	$\overline{\Delta \Sigma {XRF}_{c}}$	$\overline{\Delta {EM}_{u}}$	$\overline{\Delta {EM}_{c}}$
C	3.76 × 10^5^ ± 1.91 × 10^5^	1.36 × 10^5^ ± 1.33 × 10^5^	29.09 × 10^4^ ± 19.71 × 10^4^	2.27 × 10^4^ ± 1.45 × 10^4^
N	7.34 × 10^5^ ± 3.70 × 10^5^	1.52 × 10^5^ ± 1.35 × 10^5^	13.48 × 10^4^ ± 6.86 × 10^4^	2.29 × 10^4^ ± 1.46 × 10^4^
O	19.24 × 10^5^ ± 9.56 × 10^5^	3.99 × 10^5^ ± 3.24 × 10^5^	31.34 × 10^4^ ± 15.60 × 10^4^	5.73 × 10^4^ ± 3.65 × 10^4^

Results of the quantitative evaluation of the least squares differences of the correct and incorrect images, from the simulated images. Reported values represent average value ± standard deviation.

Lastly, the performance of the IR was investigated across different intensities for I0 (from 10^3^ to 10^10^), demonstrating a good level of robustness, with volume percentage overlap values ranging from 76 to 82%.

## Discussion

Results seem to indicate that the proposed method recovered with good fidelity, the total volume of the analyzed sample, which was attested at − 4.2% with respect to the exact sample volume (4651 μm^3^). This figure is also in line with the pixelwise average percentage difference found between the true thickness map and the one recovered iteratively after 1000 steps from the STXM map, which was − 3.4 ± 5.3%. After recovering thickness information, the IR algorithm was able to derive a 3D sample which overlapped with the original one by 82% in terms of volume with $I{R}_{os}=0.2$. It was also found that precision in the reconstruction may be increased by choosing higher values of this parameter. In this case for example, choosing $I{R}_{os}=1$ yielded a modest increase of 4% in precision.

The IR was robust against the inexact mass fractions which were derived directly from the data and deviated on average from the correct ones by 53 ± 5% for C, 15 ± 6% for N and 12 ± 8% for O, respectively. This robustness can probably be attributed to the optimization cost function, which consists of an L1 norm between the count rates of the XRF experimental image versus the ones from the simulated image, for the specific pixel being reconstructed. With this type of cost function, if the mass fractions being used are not exact, we will observe in the simulated XRF maps a displacement in intensity which renders absolute quantification challenging. On the other hand, since we are fixing the thickness and thus the available sample mass, we can expect the observed intensity displacements to have a common order of magnitude throughout a given map, following a change in the set of mass fractions. This translates into generating XRF simulated maps that have different absolute count rates from the real images but have conserved intensity proportionality among pixels within each SDD. At the same time, the intensity variation which is due to the topography variation only, while maintaining a fixed thickness, operates at different orders of magnitude and can alter the aforementioned proportionality. The IR algorithm developed for this study makes use of the intensity variation observed due to the topography variation once thickness has been fixed and attempts to derive a 3D sample by recovering the intensity proportionality among pixels rather than recovering exact count rates.

It was then verified whether the 3D reconstructed sample could be used as a way to calculate a 3D correction for self-absorption of the XRF radiation. At a first glance, the individual ${XRF}_{2D}$ images reported in Fig. 5 and the $\Sigma XRF$ images in Fig. 6a, highlighted the strong resemblance between the corrected and simulated images. This result was confirmed by the quantitative analysis reported in Table 1, where the average least squares difference in the pixelwise count rates for $\Sigma XR{F}^{c}$ resulted to be at most 1.36 × 10^5^ ± 1.33 × 10^5^ and 2.27 × 10^4^ ± 1.45 × 10^4^ for $E{M}^{c}$. Furthermore, by investigating into the $XR{F}^{s}$ of Carbon of Fig. 6a, it can be seen that the largest cell demonstrates an absorption pattern moving towards the center of the image where the three cells meet. This pattern is not observed instead in the $E{M}^{s}$ map of C in Fig. 6b, where the spherical symmetry in the signal is well conserved. This found complementarity suggests firstly that for the energies (i.e. 277 eV for the ${K}_{\alpha }$ line of C) and spatial scales involved (i.e. μm scale for cells) the absorption artefact is present and that a quantitative correction of such effect can be feasible through the presented methodology. Overall, the quantitative analysis seems to suggest that it is possible to employ the recovered 3D sample structure to numerically correct for the self-absorption effects.

The results obtained in the self-absorption correction evaluation of this study are in good qualitative agreement with the findings reported in a previous study by Malucelli et al.^19^. Namely, the recovery of the count rates in the raw XRF maps of a human cell, which are strongly asymmetric prior to the correction due to self-absorption and are modulated according to the position of their respective detector. The self-absorption correction proposed by Malucelli et al. integrates the absorption term formulated in the FP method over the entire sample’s thickness derived from the AFM, considering for each step of the integration, a single path defined by a single exit angle, traveled equally by all particles. On the other hand, the presented method exploits Ray-Tracing in an attempt to accurately quantify all the paths traveled by the particles in each different voxel source. In this way, if gaps or valleys are present between the source voxel and the detector, they are fully taken into account and do not contribute to the attenuation of the XRF radiation. In other words, we argue that a true absorption correction must be carried out at a 3D level as is suggested by Eq. (4).

In order to verify the robustness of the IR algorithm on datasets coming from different radiation sources, including ones at lower energies than synchrotrons, a series of reconstructions were carried out on the same simulated sample for a range of I0 values (between 10^3^ and 10^10^ photons). It was found that the IR works well also at lower I0 intensities (76% volume overlap with I0 = 10^3^). Furthermore, it was evaluated how varying the oversampling parameter IR_OS_ and the permutations threshold δ would affect the reconstruction time of an upscaled version of the simulated sample. This representation is exactly 2.5 times the size of the original sample and is contained in a cubic grid of 100 × 100 × 100 μm^3^. It was found that when fixing δ and using a value of 0.2 for the IR_OS,_ instead of 1.0, always yielded a 3D sample within 6% difference and up to a factor of 5 in speed gain. At the same time, varying the δ when fixing IR_OS,_ could yield a maximum gain factor of 3. By varying these two parameters together, we could find a combination (IR_OS_ = 0.2 and δ = 10^4^) that yielded a 3D sample of similar precision (85% volume overlap with simulated sample) in just above 10 min.

The presented work also offers the basis for a 3D XRF simulation framework which can be used to simulate the experimental outcome of synchrotron XRF experiments with virtually any acquisition geometry. The same framework could also be used as the basis for developing a system capable of fitting different sets of mass fractions over the STXM and XRF data provided for the IR. In this manner it would be possible in principle, after having retrieved a 3D structure through the IR, to find an optimal set of mass fractions for the fixed thickness chosen. Such a fitting system has not yet been developed and goes beyond the initial aims of this study which involve recovering a 3D sample structure and showing that 3D self-absorption correction of XRF maps can in principle be carried out through this structure. Future developments should therefore focus on the application and evaluation of the proposed framework with actual XRF datasets for which the average density is known and STXM data is available. At present, we are currently testing the IR algorithm on different types of samples that could recently be acquired and that will be the object of future works.

## Conclusion

In conclusion, through the presented IR algorithm based on ray-tracing and the use of multi-detector systems, we aim to provide a novel methodology to retrieve a 3D representation of the sample with resolved topographical landscape, from XRF data. Furthermore, we hope to have shown that starting from this sample representation it is also possible to open the way for a quantitative correction of the self-absorption artifact at the 3D level.

## Data Availability

The data that support the findings of this study are available from the corresponding author upon reasonable request.

## Code availability

We have provided a demo web application of the IR algorithm, where users can select from three simulated XRF samples and experiment with the reconstruction parameters. The web application, together with usage instructions and anonymous login, can be found at: https://github.com/ElettraSciComp/xrfstir.
